# Lifestyle and fertility-specific quality of life affect reproductive outcomes in couples undergoing *in vitro* fertilization

**DOI:** 10.3389/fendo.2024.1346084

**Published:** 2024-03-20

**Authors:** Yoko Urata, Miyuki Harada, Shinnosuke Komiya, Ikumi Akiyama, Chihiro Tuchida, Yoshiharu Nakaoka, Aisaku Fukuda, Yoshiharu Morimoto, Takuya Kawahara, Yusuke Ishikawa, Yutaka Osuga

**Affiliations:** ^1^ Department of Obstetrics and Gynecology, Faculty of Medicine, The University of Tokyo, Tokyo, Japan; ^2^ HORAC Grand Front Osaka Clinic, Osaka, Japan; ^3^ Department of Obstetrics and Gynecology, Kansai Medical University Graduate School of Medicine, Osaka, Japan; ^4^ Department of Obstetrics and Gynecology, The Fraternity Memorial Hospital, Tokyo, Japan; ^5^ IVF Namba Clinic, Osaka, Japan; ^6^ IVF Osaka Clinic, Osaka, Japan; ^7^ Clinical Research Promotion Center, The University of Tokyo Hospital, Tokyo, Japan; ^8^ FamiOne, Inc., Tokyo, Japan

**Keywords:** fertility-specific quality of life tool, FertiQoL, lifestyle, assisted reproductive technology, good-quality blastocyst rate

## Abstract

**Objective:**

A Mediterranean dietary pattern, sleeping habits, physical activity, and lifestyle appear to affect reproductive health. There are few reports about whether fertility-specific quality of life (QOL) is linked to infertility treatment outcomes. The aim of this study is to investigate when lifestyle factors and fertility-specific QOL are comprehensively considered, which factors influence assisted reproductive technology (ART) outcomes.

**Methods:**

This prospective cohort includes 291 women undergoing a first ART treatment at multiple centers in Japan and was designed to evaluate the influence of diet, physical activity, sleeping pattern, computer use duration, and fertility-specific quality of life tool (FertiQoL) score on ART treatment outcomes using a questionnaire. The primary endpoint was the good-quality blastocyst rate per oocyte retrieval and the secondary endpoints were a positive pregnancy test and gestational sac (GS) detection.

**Results:**

The good-quality blastocyst rate per oocyte retrieval tended to be negatively associated with frequent fish consumption. After all embryo transfer (ET) cycles, a positive pregnancy test tended to be positively associated with longer sleep and longer computer use (OR = 1.6, 95% CI = 0.9–2.7 and OR = 1.7, CI = 1.0–2.8, respectively) and negatively associated with a smoking partner (OR = 0.6, CI = 0.3–1.0). GS detection was positively and significantly associated with frequent olive oil intake and longer computer use (OR = 1.7, CI = 1.0–3.0 and OR = 1.7, CI = 1.0–3.0, respectively). After ET cycles with a single blastocyst, a positive pregnancy test was positively and significantly associated with longer computer use (OR = 2.0, CI = 1.1–3.7), while GS detection was significantly more likely in women with longer computer use (OR = 2.1, CI = 1.1–3.8) and tended to be more likely in women with a higher FertiQoL Total scaled treatment score (OR = 1.8, CI = 1.0–3.3). p < 0.05 was considered statistically significant and 0.05 ≤ p <0.01 as tendency.

**Conclusions:**

Olive oil may be an important factor in dietary habits. Fertility-specific QOL and smoking cessation guidance for partners are important for infertile couples.

## Introduction

Infertility is defined as the failure to achieve a successful pregnancy after 12 months or longer of regular unprotected intercourse and is reported to affect approximately 48.5 million couples in the world (1). The number of infertile patients is increasing (2). According to the International Committee for Monitoring Assisted Reproductive Technologies, the number of assisted reproductive technology (ART) treatment cycles is gradually increasing (3) and is reported to be about 2.5–3 million cycles per year worldwide (https://www.icmartivf.org/wp-content/uploads/ICMART-ESHRE-WR2018-Preliminary-Report.pdf) (4). In Japan, 449,900 ART cycles are performed annually (https://www.jsog.or.jp/activity/art/2020data_202208.pdf) and approximately 1 in 13.9 babies is born as a result of ART. In Japan, infertility treatment, including ART, has been covered by public health insurance since April 2022. The number of couples undergoing infertility treatment is increasing due to aging of the population who wish to have children owing to later marriages, the expansion of women’s participation in society and diversification of their life plans, and the growing need for more planned pregnancies and deliveries.

Some factors affect infertility treatment results, including age and ovarian reserve, which can be evaluated by the anti-Müllerian hormone (AMH) level, follicle-stimulating hormone (FSH) level, and antral follicle count. In addition, lifestyle factors are reportedly associated with fecundity and infertility treatment results, including dietary habits such as consumption of seafood (5), a Mediterranean diet (6, 7), and intake of alcohol (8, 9) and caffeine (10); smoking (11, 12); exercise habits (13, 14); and the quality and duration of sleep (15, 16). However, it has not been fully elucidated whether lifestyle factors are associated with infertility.

Quality of life (QOL) was defined by the World Health Organization (WHO) as ‘‘individuals’ perceptions of their position in life in the context of the culture and value systems in which they live and in relation to their goals, expectations, standards, and concerns’’ (17). QOL must be assessed from both general and disease-specific perspectives (18). Scales used for infertility-specific QOL assessment include the Fertility Quality of Life tool (FertiQoL) and Fertility Problem Inventory (18, 19). FertiQoL contains a Core module and an additional module for assessment of treatment satisfaction (19), is available in 40 or more languages including Japanese (20, 21), and is an easy tool to use around the world. In this study, we downloaded the Japanese version from the FertiQoL homepage (https://sites.cardiff.ac.uk/fertiqol/). FertiQoL has been used to objectively assess patient satisfaction (22, 23) and to assess QOL of patients undergoing infertility treatment (24, 25) and patients with polycystic ovary syndrome (26). However, there are few reports about whether patients’ QOL evaluated by FertiQoL is linked to infertility treatment outcomes.

The purpose of this study was to elucidate the impact of lifestyle, including dietary habits and physical activity, and fertility-specific QOL on ART outcomes by performing a comprehensive analysis.

## Materials and methods

### Recruitment of patients and inclusion and exclusion criteria

Infertile couples seeking their first IVF treatment at three clinics of the IVF Japan Group in Osaka, Japan, and the University of Tokyo Hospital in Tokyo, Japan, were recruited to participate in this prospective cohort study. Female partners aged 20–45 years who had a body mass index (BMI) ≥ 18 and < 30 kg/m^2^, had an AMH level ≥ 1 ng/mL, and who used their own oocytes and male partners who used their own sperm were eligible. Women are of Eastern Asian ethnicity living in Japan; with endometrioma ≥ 3 cm; who had a history of neoplasm, diabetes mellitus, or hypertension; who were treated with recombinant follicle-stimulating hormone (rFSH) or human menopausal gonadotropin (hMG) at a dosage ≥ 450 IU/day; or who were psychiatric outpatients were excluded.

### Frequencies of food consumption and social habits and FertiQoL questionnaire

At their first visit to the IVF unit, we recruited patients to this prospective cohort study and gave them the questionnaire. The food categories investigated were principally based on the Mediterranean diet score (27), which was modified for adjustment to Japanese dietary habits, and included refined cereals (white rice, bread, and pasta), non-refined cereals (brown and cereal rice), potatoes, fruits, vegetables, legumes (natto and tofu), fish, red meat and products, poultry, full-fat dairy products (e.g., cheese, yoghurt, and milk), olive oil in cooking, alcoholic beverages, caffeine-containing drinks (coffee, black tea, green tea, etc.), and breakfast. The lifestyle categories investigated were sleep duration, work duration, night shift or not, computer use duration (computer, smartphone, and tablet), and smoking habits of the patient and her partner. The degree of smoking was evaluated using the Brinkman Index, which multiplies the duration of smoking (in years) by the number of cigarettes smoked per day (28). Exercise habits were investigated based on the WHO’s definition of exercise intensity using a table of the metabolic equivalent of task (MET) according to physical activity: sedentary behavior (1.5 METs or lower), light-intensity physical activity (1.5–3 METs), moderate-intensity physical activity (between 3 and <6 METs), and vigorous-intensity physical activity (6.0 or more METs) (29, 30). The FertiQoL questionnaire in Japanese was downloaded from the FertiQoL homepage (https://sites.cardiff.ac.uk/fertiqol/).

### Controlled ovarian stimulation, laboratory procedures, and embryo transfer

For the long protocol regimen, nasal administration of a gonadotropin-releasing hormone (GnRH) agonist (buserelin acetate, Suprecur; Clinigen, Tokyo, Japan) was started in the middle of the luteal phase prior to ovarian stimulation. Controlled ovarian stimulation was started on cycle day 2 or 3 with oral Clomifene citrate (Clomid; Fuji Pharma, Toyama, Japan), an aromatase inhibitor (Letrozole, Femara; Novartis, Tokyo, Japan), rFSH (follitropin alfa, Gonal-F; Merck, Tokyo, Japan), and hMG (HMG; ASKA Pharma, Tokyo, Japan). An individual starting drug at an individual dosage was administered based on the AMH level, age, and basal FSH level. The allowed maximum dosages of rFSH and hMG were 300 IU/day. The dosage could be adjusted according to the general practice of the participating clinics and hospital. For the GnRH antagonist protocol regimen, a GnRH antagonist (ganirelix acetate, GANIREST; Organon, Tokyo, Japan) at a dosage of 0.25 mg/day was added on the day when the leading follicle had a mean diameter larger than 14 mm and was continued throughout the remaining stimulation. Final oocyte maturation was induced by administering 0.5 mg of a GnRH agonist (nafarelin acetate hydrate, Nasanyl; Pfizer, Tokyo, Japan) or 10,000 IU of human chorionic gonadotropin (hCG) (HCG; Mochida Pharmaceutical Co., Tokyo, Japan). Oocyte retrieval was performed 34 hours after GnRH agonist or hCG administration. Fertilized oocytes were cultured to the cleavage or blastocyst stage, which was assessed according to the classification system of Veeck or the Gardner classification, respectively, and vitrificated. Blastocysts with a Gardner score of 3BB or greater were considered to be good quality. Blastocysts were ranked based on morphological evaluation so that the blastocyst with the highest implantation potential was used first.

A wash-out period of at least one completed menstrual cycle was required between stimulation and ET. Endometrial preparation was performed in a hormone replacement cycle. Transdermal administration of estradiol (ESTRANA; Hisamitsu Pharma, Saga, Japan) began on day 2 or 3 of the menstrual cycle. When endometrial thickness reached at least 8 mm, a vaginal progesterone tablet (LUTINUS; Ferring Pharma, Tokyo, Japan) at a dosage of 300 mg/day was added. Cleavage- or blastocyst-stage embryos were thawed and transferred on day 3 or 5, respectively, after commencement of progesterone. For two-step ET, cleavage- and blastocyst-stage embryos were thawed and transferred on day 3 and 5, respectively, after commencement of progesterone during a single ET cycle. The number of embryos transferred was decided based on the general practice of the participating clinics and hospital.

A serum hCG test was conducted generally between 3 weeks 6 days and 4 weeks 1 day of gestation. Providing the test was positive, transvaginal ultrasound was performed 1 or 2 weeks later to detect an intrauterine gestational sac (GS).

### Reproductive outcomes

Serum levels of hormones, including FSH, luteinizing hormone, prolactin, and AMH, before the ART cycle were determined. The outcomes of IVF/intracytoplasmic sperm injection (ICSI) were recorded, including the number of follicles larger than 15 mm and the estradiol level when oocytes were picked up, the number of retrieved oocytes, the fertilization rate, and the numbers of cleavage-stage embryos, blastocysts, and good-quality blastocysts. A good-quality blastocyst was defined as a blastocyst with a score of 3BB or greater based on the Gardner classification. The result of the first ET cycle after oocyte retrieval was assessed. A positive pregnancy test was defined as a serum hCG level ≥10 mIU/mL, generally measured between 3 weeks 6 days and 4 weeks 1 day of gestation. The fertilization rate was calculated as the number of two-pronuclear embryos relative to the number of retrieved oocytes, the achievement rate of the cleavage stage was calculated as the number of cleavage-stage embryos relative to the number of retrieved oocytes, and the good-quality blastocyst rate was calculated as the number of good-quality blastocysts relative to the number of retrieved oocytes.

### Statistics

We aimed to recruit 286 patients in order to detect a 5% difference in the good-quality blastocyst rate, which we considered a clinically relevant difference, between two groups defined by independent variables, with a two-sided alpha error of 5% and power of 80%. As independent variables, analysis was conducted to determine if background, dietary habits, exercise habits, and FertiQoL were related to IVF outcomes. For continuous variables, women were divided into two groups (higher and lower than the median value) and the two groups were compared. The primary outcome was the good-quality blastocyst rate per oocyte retrieval. Secondary outcomes were a positive pregnancy test (hCG level ≥ 10 mIU/mL) and detection of a GS. We screened variables associated with each of these outcomes using the two-sample t-test with a threshold of p < 0.01 (univariate selection (31)) to reduce the number of independent variables). The associated variables were included in the multivariable model. A multivariable linear model was used for the good-quality blastocyst rate and multivariable logistic models were used for a positive pregnancy test and detection of a GS. In the resulting model, independent variables with p < 0.05 were considered to be significantly associated with the outcome. Missing data were not imputed. All statistical analyses were performed with SAS (version 9.4; SAS Institute Inc., Cary, NC, USA).

## Results

### Study patients

**Figure 1:**
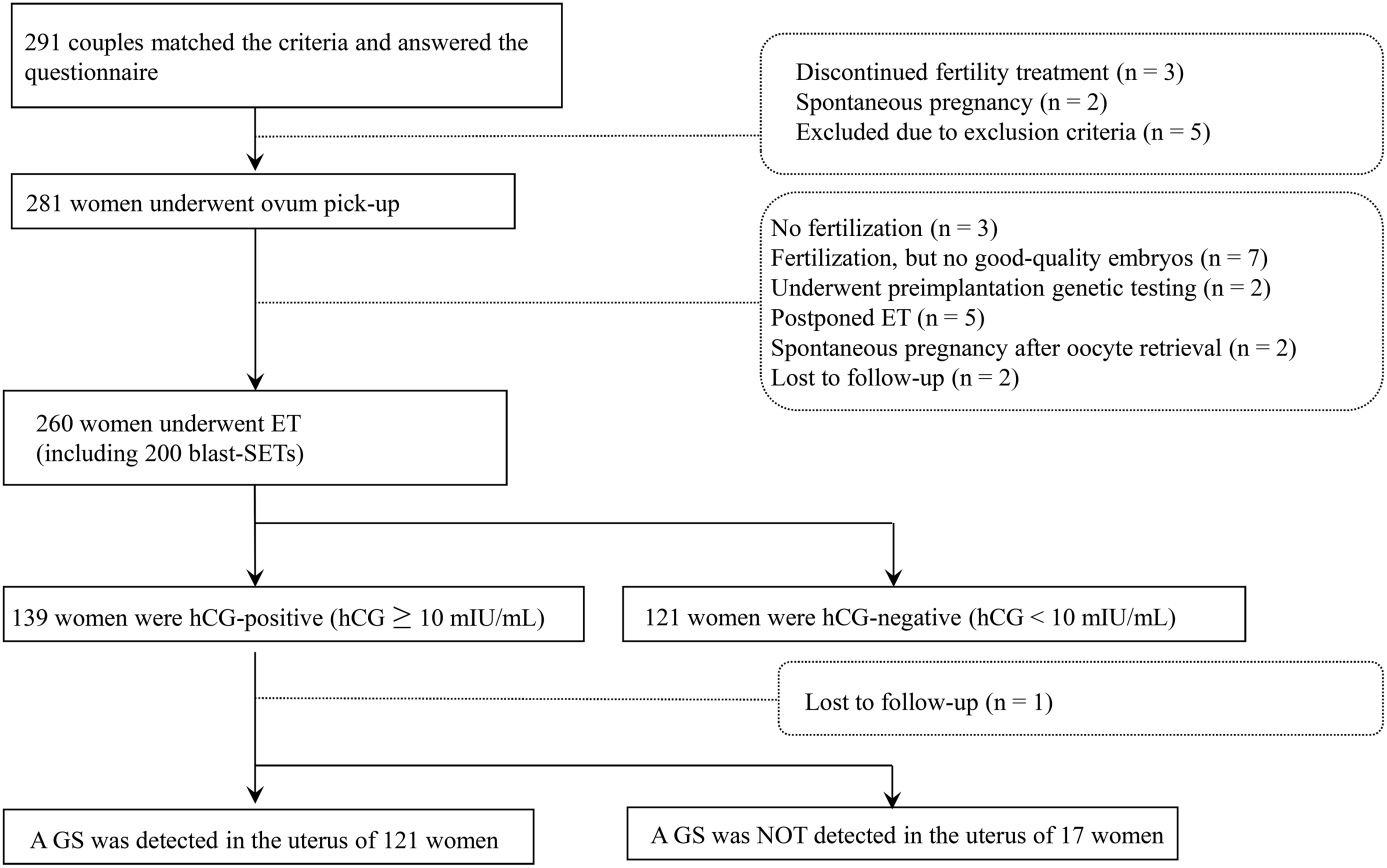
Flowchart of *in vitro* fertilization treatment of the study population.

From May 2019 to March 2022, 291 women met the eligibility criteria and provided informed consent. Of these women, three discontinued fertility treatment, two spontaneously became pregnant before controlled ovarian stimulation, and five met the exclusion criteria. Therefore, 281 women underwent controlled ovarian stimulation and oocyte retrieval. Of these women, three did not achieve fertilization, seven did not produce any good-quality embryos, two underwent preimplantation genetic testing, five postponed their ET, two spontaneously became pregnant before ET, and two were lost following treatment. Therefore, 260 women underwent ET including 200 single blastocyst embryo transfers (blast-SETs), 139 women showed hCG positivity (hCG level ≥ 10 mIU/mL), and a single GS was detected in 121 women. No multiple GSs were observed (**Figure 1**). The characteristics, dietary habits, lifestyle characteristics, and FertiQoL scores of patients at baseline are provided in **Table 1**. The clinical characteristics of patients upon IVF treatment are provided in **Table 2**.

**Table 1:** Patients demographic, clinical and lifestyle characteristics, dietary habits and FertiQoL score.

		median	range	Number	%
Demographic characteristics					
Age (years)		37	(21-45)		
BMI (kg/m2)		20.8	(18.0-29.7)		
	<18.5			21	7.5
	18.5-24.99			232	82.6
	≧25			28	10.0
Clinical characteristics					
FSH (mIU/mL)		7.3	(1.7-43.2)		
LH (mIU/mL)		5.1	(0.5-15.1)		
PRL (ng/mL)		14.6	(0.25-100)		
AMH (ng/mL)		2.73	(0.31-13.5)		
Complication	Yes			119	42.3
	No			162	57.7
Fibroid	Yes			32	11.4
	No			249	88.6
Asthma	Yes			2	0.7
	No			279	99.3
Hyperthyroidism	Yes			7	2.5
	No			274	97.5
Hashimoto's disease	Yes			15	5.3
	No			266	94.7
Infertility factor					
	Female			45	16.0
	Male			37	13.2
	Both			9	3.2
	Unknown			190	67.6
Dietary habits					
Refined cereals	times/week	10	(0-27)		
Non-refined cereals	times/week	0	(0-21)		
Potatoes	times/week	1	(0-7)		
Fruits	times/week	2	(0-21)		
Vegetables	times/week	7	(2-23)		
Legumes	times/week	5	(0-18)		
Fish	times/week	2	(0-14)		
Red meat and products	times/week	5	(0-21)		
Poultry	times/week	3	(0-16)		
Full-fat dairy products (cheese, yoghurt, milk)	times/week	7	(0-21)		
Olive oil in cooking	times/week	3	(0-20)		
Alcoholic beverages	Yes			125	
	No			155	
	units/week	0	(0-19.6)		
Caffeine-containing drinks	times/week	7	(0-50)		
Breakfast	times/week	7	(0-7)		
Lifestyle characteristics					
Sleep duration	hr/day	7	(4-10)		
Work duration	hr/week	10	(0-195)		
Night shift					
	Yes			88	31.3
	No			191	68.0
Computer use duration	hr/day	4	(0.5-20)		
Patient's smoking history					
	Never			220	78.3
	Former			54	19.2
	Current			7	2.5
	Brinkman index	0	(0-510)		
Partner age		37	(25-56)		
Partner's smoking history					
	Never			143	50.9
	Former			61	21.7
	Current			76	27.0
	Brinkman index	44.5	(0-780)		
Exercise habits					
Vigorous-intensity physical activity	min/day	0	(0-360)		
Moderate-intensity physical activity	min/day	0	(0-300)		
Light-intensity physical activity	min/day	30	(0-600)		
Sedentary behavior	min/day	360	(0-1200)		
FertiQoL score					
Scaled subscale: Emotional		54.2	(0-100)		
Scaled subscale: Mind/body		62.5	(4.1-100)		
Scaled subsclae: Relational		75.0	(16.6-100)		
Scaled subscale: Social		66.7	(12.5-100)		
Scaled subscale: Environment		62.5	(16.6-100)		
Scaled subscale: Tolerability		50.0	(0-100)		
Total scaled core score		63.0	(18.7-95.8)		
Total scaled treatment score		57.5	(17.5-97.5)		
Total scaled FertiQoL score		61.8	(27.9-93.3)		

BMI, body mass index.

FSH, follicle-stimulating hormone.

LH, luteinizing hormone.

PRL, prolactin.

AMH, anti-mullerian hormone.

**Table 2:** Clinical characteristics.

		median	range	Number	%
Cycle-specific clinical characteristics (n=281, IVF/ICSI cycles)					
Controlled ovarian stimulation					
	Short			12	4.3
	Long			100	35.6
	Antagonist			152	54.1
	Mild			17	6.0
Insemination technique					
	IVF			81	28.8
	ICSI			156	55.5
	Split			44	15.7
Total gonadotropin (hMG, FSH)		1875	(0-5775)		
The number of follicles at oocyte retrieval (15 mm and larger)		7	(1-25)		
Estradiol level at oocyte retrieval		2481	(115.2-13224		
The number of eggs		12	(1-41)		
The number of fertilization		8	(0-28)		
The number of cleavage-stage embryos		7	(0-28)		
The rate of cleavage stage embryo per fertilization		100	(14.28-100)		
The number of good-quality blastocysts		2	(0-15)		
The number of blastocysts		3.5	(0-19)		
The rate of good quality-blastocyst per oocyte retrieval		17.39	(0-100)		
Embryo transfer					
	Yes			260	
	Cleavage-SET			43	16.5
	Blast-SET			200	76.9
	Cleavage-DET			8	3.1
	Blast-DET			1	0.4
	2 step			8	1.9
	No			21	
Cycle-specific clinical characteristics (n=260, ET cycles)					
Positive pregnancy test	Yes			139	53.5
	No			121	46.5
GS-positive	Yes			121	46.5
	No			138	53.1
	Unknown			1	0.4
FHB	Yes			104	40.0
	No			154	59.2
	Unknown			2	0.8

IVF, *in vitro* fertilization.

ICSI, intracytoplasmic sperm injection.

Split, *in vitro* fertilization and intracytoplasmic sperm injection.

hMG, human menopausal gonadotropin.

FSH, follicle-stimulating hormone.

SET, single embryo transfer.

DET, double embryo transfer.

ET, embryo transfer.

GS, gestational sac.

FHB, fetal heart beat.

### The good-quality blastocyst rate per oocyte retrieval

Women aged 36 years or older had a significantly lower good-quality blastocyst rate per oocyte retrieval than those younger than 36 years (p = 0.007), and women with Hashimoto’s disease had a significantly lower good-quality blastocyst rate per oocyte retrieval than those without it (p = 0.004) (**Table 3**). Women who consumed fish twice/week or more tended to have a lower good-quality blastocyst rate per oocyte retrieval than those who consumed fish less often (p = 0.065).

**Table 3:** The good-quality blastocyst rate per oocyte retrieval (n = 270).

Variable		Reference	Estimate (SE)	p value
Fish intake	High	Low	-4.5 (2.4)	0.065
Caffeine intake	High	Low	-3.4 (2.2)	0.124
Working status	Full-time	None	-0.1 (3.3)	0.975
	Part-time	None	2.5 (2.9)	0.378
Age	≥ 36 years	< 36 years	-6.1 (2.2)	0.007
Hashimoto’s disease	Yes	No	-14.2 (4.9)	0.004

SE, standard error.

Higher fish intake = twice/week or more.

Higher caffeine intake = seven times/week or more.

### Positive pregnancy test after all ET cycles

We analyzed factors associated with a positive pregnancy test after all ET cycles, including single embryo transfer (SET) (blastocyst or cleavage stage), double embryo transfer (DET) (blastocyst or cleavage stage), and two-step ET (one blastocyst and one cleavage-stage embryo) (**Table 4**). Women aged 36 years or older were significantly less likely to have a positive pregnancy test than those younger than 36 years (odds ratio (OR) = 0.4, 95% confidence interval (CI) = 0.3–0.8, p = 0.003). Women who slept for 7 hours/day or longer and used a computer for 4 hours/day or longer tended to be more likely to have a positive pregnancy test (OR = 1.6, 95% CI = 0.9–2.7, p = 0.088; and OR = 1.7, 95% CI = 1.0–2.8, p = 0.059, respectively). Women whose partners were smokers tended to be less likely to have a positive pregnancy test than women whose partners were non-smokers (OR = 0.6, 95% CI = 0.3–1.0, p = 0.063).

**Table 4:** Positive pregnancy test after all embryo transfer cycles (n = 254).

Variable		Reference	OR (95% CI)	p value
Daily sleep	Longer	Shorter	1.6 (0.9–2.7)	0.088
Computer use	Longer	Shorter	1.7 (1.0–2.8)	0.059
Smoking (partner)	Yes	No	0.6 (0.3–1.0)	0.063
Age	≥ 36 years	< 36 years	0.4 (0.3–0.8)	0.003

OR, odds ratio.

CI, confidence interval.

Longer sleep = 7 hours/day or longer.

Longer computer use = 4 hours/day or longer.

### GS detection after all ET cycles

We analyzed factors associated with GS detection after all ET cycles (**Table 5**). A GS was significantly more likely to be detected in women who took olive oil three times/week or more, used a computer for 4 hours/day or longer, and whose BMI was 20.8 kg/m^2^ or higher (OR = 1.7, 95% CI = 1.0–3.0, p = 0.041; OR = 1.7, 95% CI = 1.0–3.0, p = 0.039; and OR = 1.8, 95% CI = 1.1–3.1, p = 0.031, respectively). A GS was significantly less likely to be detected in women aged 36 years or older than in those younger than 36 years (OR = 0.3, 95% CI = 0.2–0.5, p < 0.001).

**Table 5:** Gestational sac detection after all embryo transfer cycles (n = 254).

Variable		Reference	OR (95% CI)	p value
Olive oil intake	High	Low	1.7 (1.0–3.0)	0.041
Computer use	Longer	Shorter	1.7 (1.0–3.0)	0.039
Age	≥ 36 years	< 36 years	0.3 (0.2–0.5)	<0.001
BMI	≥ 20.8 kg/m^2^	< 20.8 kg/m^2^	1.8 (1.1–3.1)	0.031

OR, odds ratio.

CI, confidence interval.

BMI, body mass index.

High olive oil intake = three times/week or more.

Higher olive oil intake = three times a week or more.

Longer computer use = 4 hours/day or longer.

### Positive pregnancy test after single blastocyst embryo transfer cycles

To remove the influence of the number and type of embryos transferred, we evaluated factors associated with a positive pregnancy test after blast-SET cycles only (**Table 6**). Women who used a computer for 4 hours/day or longer were significantly more likely to have a positive pregnancy test (OR = 2.0, 95% CI = 1.1–3.7, p = 0.017). Women aged 36 years or older tended to be less likely to have a positive pregnancy test (OR = 0.6, 95% CI = 0.3–1.0, p = 0.055).

**Table 6:** Positive pregnancy test after single blastocyst embryo transfer cycles (n = 199).

Variable		Reference	OR (95% CI)	p value
Computer use	Longer	Shorter	2.0 (1.1–3.7)	0.017
Age	≥ 36 years	< 36 years	0.6 (0.3–1.0)	0.055

OR, odds ratio.

CI, confidence interval.

Longer computer use = 4 hours/day or longer.

### GS detection after blast-SET cycles

We analyzed factors associated with GS detection after blast-SET cycles only (**Table 7**). A GS was significantly more likely to be detected in women who used a computer for 4 hours/day or longer (OR = 2.1, 95% CI = 1.1–3.8, p = 0.016). A GS was significantly less likely to be detected in women aged 36 years or older (OR = 0.4, 95% CI = 0.2–0.8, p = 0.005). GS detection tended to be more likely in women with a higher FertiQoL Total scaled treatment score and a BMI of 20.8 kg/m^2^ or higher (OR = 1.8, 95% CI = 1.0–3.3, p = 0.059; and OR = 1.7, 95% CI = 0.9–3.1, p = 0.080, respectively).

**Table 7:** Gestational sac detection after single blastocyst embryo transfer cycles (n = 196).

Variable		Reference	OR (95% CI)	p value
Computer use	Longer	Shorter	2.1 (1.1–3.8)	0.016
FertiQoL Total scaled treatment score	High	Low	1.8 (1.0–3.3)	0.059
Age	≥ 36 years	< 36 years	0.4 (0.2–0.8)	0.005
BMI	≥ 20.8 kg/m^2^	< 20.8 kg/m^2^	1.7 (0.9–3.1)	0.080

OR, odds ratio.

CI, confidence interval.

FertiQoL, fertility-specific quality of life.

BMI, body mass index.

Longer computer use = 4 hours/day or longer.

High FertiQoL Total scaled treatment score = 57.5 or higher.

## Discussion

This study comprehensively examined whether background factors, lifestyle, dietary habits, and fertility-specific QOL are related to ART outcomes in female partners of Eastern Asian ethnicity using a questionnaire before the first cycle of ART. Advanced age and Hashimoto's disease were negatively and significantly associated with the good-quality blastocyst rate per oocyte retrieval, while greater fish consumption tended to be negatively associated with this rate. When all ET cycles were evaluated, longer sleep and longer computer use were positively associated with a positive pregnancy test, advanced age was negatively and significantly associated with a positive pregnancy test, and a partner who was a smoker was negatively associated with a positive pregnancy test. GS detection was positively and significantly associated with more frequent olive oil intake, longer computer use, and a higher BMI, but negatively and significantly associated with advanced age. When only blast-SET cycles were evaluated to exclude the effects of the number and type of embryos, longer computer use was positively and significantly associated with a positive pregnancy test, while advanced age tended to be negatively associated with a positive pregnancy test. Longer computer use was positively and significantly associated with GS detection, a higher FertiQoL Total scaled treatment score and higher BMI tended to be positively associated with GS detection, and advanced age was negatively and significantly associated with GS detection.

Advanced age negatively affects ovarian reserve (32) and implantation (33). Furthermore, autoimmune diseases including thyroid disorders cause primary ovarian insufficiency (34), and Hashimoto’s disease adversely affects ovarian function. Consistently, in the current study, older age had a significant negative impact on the good-quality blastocyst rate per oocyte retrieval, a positive pregnancy test after all ET cycles, and GS detection after all ET and blast-SET cycles. Women with Hashimoto’s disease had a significantly lower good-quality blastocyst rate per oocyte retrieval.

It has been reported that female obesity (BMI ≥ 30 kg/m^2^) may adversely affect IVF outcomes. In a report comparing four groups (obese, overweight, normal weight, and underweight), the numbers of oocytes retrieved and blastocysts did not differ among the groups. However, the rates of implantation and pregnancy were decreased in the obese group, while the implantation rate was decreased and the pregnancy rate was unchanged in the underweight group (BMI < 20 kg/m^2^) (35). Hu et al. reported that the cumulative number of live births after IVF/ICSI was significantly higher in the normal BMI group (18.5–24.9 kg/m^2^) than in the high BMI group (≥ 25 kg/m^2^) (36). However, in that study, the inclusion criteria were 18 ≤ BMI < 30 kg/m^2^, which means that the high and low BMI groups were defined as 20.8 ≤ BMI < 30 kg/m^2^ and 18 ≤ BMI < 20.8 kg/m^2^, respectively, which is inconsistent with the WHO’s criteria for obesity and underweight. Therefore, it is difficult to compare our results with those of previous reports. This study suggests that a lower BMI (18 ≤ BMI < 20.8 kg/m^2^), even if an individual is not underweight, may negatively impact ART results.

Although there are a relatively large number of reports about dietary habits including consumption of seafood and fish, there are no consistent reports about whether seafood consumption positively affects fertility (5, 37–39). The reason for these inconsistent results may be that seafood contains both fertility-enhancing and -damaging substances. Seafood is an important source of omega-3 fatty acids, which are important for steroidogenesis (40, 41) and have anti-inflammatory effects (42). Additionally, among 235 women undergoing IVF/ICSI treatment, omega-3 intake improved embryo morphology (43). However, seafood also contains persistent organic pollutants, such as polychlorinated biphenyls (44, 45) and mercury (46).

A Mediterranean diet, which includes olive oil, was reported to increase the rates of implantation, clinical pregnancy, and live births in a prospective study (7). In addition, intake of olive oil, vitamin D, and marine omega-3 fatty acids for 6 weeks improved embryo quality (47). Neither of these reports evaluated the influence of olive oil alone. The present study demonstrated that olive oil intake alone increased GS detection after all ET cycles. The most important component of a Mediterranean diet for improvement of fertility was unclear in previous reports. This study suggested that olive oil may be the most important contributor.

The quality and duration of sleep have been reported to affect infertility treatment outcomes (48), and Goldstein et al. reported that there tended to be a correlation between sleep duration and the number of oocytes retrieved in IVF treatment (49). In addition, Yao et al. divided sleep duration into five groups: less than 7 hours, 7–8 hours, 8-9 hours, and 9-10 hours and 10 hours or more. They reported that the numbers of eggs retrieved and metaphase II oocytes were 11.5% and 11.9% lower, respectively, among women who slept for less than 7 hours compared with those who slept for 7–8 hours. The clinical pregnancy rate was lower among women who slept for 9–10 hours than among those who slept for 7–8 hours (OR = 0.65). In a cohort of general women who wanted to have children, women who slept for 9 or more hours showed longer time to pregnancy than those who slept for 8 hours (15). In this study, the median sleeping duration was 7 hours and only 2.1% of women (6 of 281 women) slept for 9 hours or longer, perhaps because Japanese women sleep for shorter durations than women from other countries. Our study analyzed data using only a two-group comparison, and the results are consistent with previous reports because women who slept for 7 hours or longer tended to have a higher possibility of a positive pregnancy test after all ET cycles than those who slept for less than 7 hours.

There are reports that evening use of light-emitting eReaders negatively affects sleep (50) and that the quality and duration of sleep affect IVF outcomes (16, 48). There are no reports about the relationship between use of computers and light-emitting eReaders and IVF outcomes. In this study, prolonged computer use was positively associated with a positive pregnancy test and GS detection, was significantly correlated with intake of non-refined cereals once per week or more (correlation coefficient = 0.128, p = 0.0313), working longer (correlation coefficient = 0.16232, p = 0.0069), and sitting longer (correlation coefficient 0.26595, p < 0.0001), and tended to be positively correlated with a higher BMI (correlation coefficient = 0.10367, p = 0.0828). Based on the correlation with intake of non-refined cereals, long computer users may be more health-conscious. The correlations with longer working and sitting suggest that long computer users are long-time workers who use computers in a sitting position, and sitting for a long time may increase BMI. GS detection after all ET cycles significantly correlated with a higher BMI in this study; therefore, the higher possibility of GS detection among long computer users may be due to BMI.

The effects of male smoking on fertility have been previously examined, with worse semen findings (volume, concentration, and total sperm number) among smokers (51). Male smokers had a 2.4% lower possibility of achieving a 12-week pregnancy with every 1-year increase in age (52). However, there are inconsistent results about the effects of male smoking on assisted reproduction. Borges et al. reported that male smokers had lower fertilization and blastocyst formation rates and unchanged implantation rates (51). Hoek et al. demonstrated that among 490 ICSI embryos from 113 women and 41 men, embryo morphology assessed using a time-lapse morphokinetic selection algorithm (KIDScore) was significantly and negatively affected by male smoking (53). On the other hand, Frappier et al. reported no difference in IVF outcomes in 252 couples undergoing IVF with a male smoking history (54). Although semen findings were not examined in the current study, the positive pregnancy test rate after all ET cycles tended to be lower among women whose partners were smokers. This suggests that male smoking negatively impacts ART outcomes.

Some meta-analyses assessed the relationship between stress and distress and reproductive outcomes of ART (55, 56). However, few reports demonstrate the correlation between infertility treatment outcomes and fertility-specific QOL such as FertiQoL. Santoro et al. reported that among women undergoing infertility treatment without ART, the Emotional FertiQoL score was positively related to pregnancy and a singleton live birth in women with polycystic ovary syndrome, while the Mind/Body FertiQoL score was negatively associated with a singleton live birth in women with unexplained infertility. The results were inconsistent between these two groups of women. Therefore, Santoro concluded that FertiQoL scores were not significant factors to estimate pregnancy outcomes (26). In disagreement with previous reports, in the current study, women with a higher FertiQoL Total scaled treatment score tended to have a higher possibility of GS detection after blast-SET. The FertiQoL score consists of Core subscales containing four factors (emotional, mind-body, relational, and social) and Treatment subscales containing two factors (environment and treatment tolerability) (20, 21). The FertiQoL Total scaled treatment score indicates impacts related to treatment environment (e.g., access, quality, and interactions with staff) and impacts due to consequences of treatment (e.g., physical and mode effects, and daily disruptions). A higher treatment score indicates more comfortable treatment. This study implies that a GS tended to be detected in women with more comfortable treatment after blast-SET. In addition, a meta-analysis including 39 reports (n = 2746) demonstrated that psychosocial interventions improved psychological distress and increased clinical pregnancy; however, it did not evaluate the psychological condition using a fertility-specific QOL scale such as FertiQoL or identify which type of distress is a contributor (57). Domar et al. reported that among women undergoing IVF treatment, brief self-administered cognitive coping and relaxation intervention improved Core and Treatment FertiQoL scores, but not the clinical pregnancy rate (58). In the current study, FertiQoL was only assessed prior to treatment; therefore, further investigations are needed to determine whether interventions to improve Treatment FertiQoL scores improve infertility treatment outcomes.

A strength of this study is that a detailed and comprehensive questionnaire including detailed dietary habits with reference to the Mediterranean diet score and fertility-specific QOL using FertiQoL was completed before the first cycle of ART treatment, and its results were compared with subsequent ART outcomes, namely, the first cycle of oocyte retrieval and ET. Consequently, we studied a homogeneous population with little bias. The weaknesses of the study are the small number of cases and the lack of detailed evaluation of dietary contents, such as intakes of individual nutrients and results of fetal heart beat positivity and live birth.

Our results have some implications for clinical practice. In accordance with previous reports, longer sleep was associated with better ART outcomes, but it is unclear whether much longer sleep is better. Longer computer use was not necessarily negative for ART outcomes. More frequent fish consumption had a possible negative effect on the good-quality blastocyst rate per oocyte retrieval. While dietary habits including consumption of olive oil have been reported to positively affect fertility treatment, this is the first time that olive oil intake alone has been reported to have a positive effect. For the first time, this study suggests that a partner’s smoking negatively impacts a couple’s infertility treatment outcomes and that smoking cessation guidance for partners is important for infertile couples. Although it is unclear whether interventions to improve fertility-specific QOL affect treatment efficacy, it is important to understand that fertility-specific QOL may influence ART outcomes in clinical practice.

In summary, our findings suggest that long computer use does not necessarily have a negative effect on ART outcomes and that olive oil intake may be the most important dietary habit for improvement of ART outcomes. Further investigation is required to elucidate the influence of fish consumption, a partner’s smoking, and fertility-specific QOL on ART outcomes.

## Data availability statement

The original contributions presented in the study are included in the article/supplementary material. Further inquiries can be directed to the corresponding author.

## Ethics statement

The studies involving humans were approved by The institutional ethics committee of the University of Tokyo. The studies were conducted in accordance with the local legislation and institutional requirements. The participants provided their written informed consent to participate in this study.

## Author contributions

YU: Data curation, Writing – original draft, Writing – review & editing, Funding acquisition, Investigation, Project administration. MH: Data curation, Investigation, Writing – original draft, Writing – review & editing, Conceptualization, Methodology, Supervision. SK: Data curation, Writing – review & editing. IA: Methodology, Writing – review & editing, Data curation. CT: Data curation, Writing – review & editing. YN: Writing – review & editing. AF: Writing – review & editing. YM: Conceptualization, Writing – review & editing. TK: Formal analysis, Writing – original draft, Writing – review & editing. YI: Conceptualization, Writing – review & editing. YO: Writing – review & editing, Conceptualization.
